# An integrative multi-omics modeling framework for chemotherapy response prediction through microRNA–target regulatory signatures

**DOI:** 10.1093/bib/bbag560

**Published:** 2026-10-13

**Authors:** Phuong Lam Tran, Chi-Nga Chow, Yun Tang, Michael Anekson Widjaya, You Sheng Paik, Yann-Jen Ho, Wei-Chih Huang, Chia-Ru Chung, Wen-Chi Chang, Muh-Hwa Yang, Tzong-Yi Lee

**Affiliations:** Institute of Bioinformatics and Systems Biology, College of Engineering Bioscience, National Yang Ming Chiao Tung University, No. 75, Boai Street, East Dist., Hsinchu City 300, Taiwan; Institute of Bioinformatics and Systems Biology, College of Engineering Bioscience, National Yang Ming Chiao Tung University, No. 75, Boai Street, East Dist., Hsinchu City 300, Taiwan; Institute of Bioinformatics and Systems Biology, College of Engineering Bioscience, National Yang Ming Chiao Tung University, No. 75, Boai Street, East Dist., Hsinchu City 300, Taiwan; Institute of Bioinformatics and Systems Biology, College of Engineering Bioscience, National Yang Ming Chiao Tung University, No. 75, Boai Street, East Dist., Hsinchu City 300, Taiwan; Institute of Bioinformatics and Systems Biology, College of Engineering Bioscience, National Yang Ming Chiao Tung University, No. 75, Boai Street, East Dist., Hsinchu City 300, Taiwan; Institute of Bioinformatics and Systems Biology, College of Engineering Bioscience, National Yang Ming Chiao Tung University, No. 75, Boai Street, East Dist., Hsinchu City 300, Taiwan; Institute of Bioinformatics and Systems Biology, College of Engineering Bioscience, National Yang Ming Chiao Tung University, No. 75, Boai Street, East Dist., Hsinchu City 300, Taiwan; Department of Computer Science and Information Engineering, National Central University, No. 300, Zhongda Rd., Zhongli Dist., Taoyuan City 320317, Taiwan; Institute of Tropical Plant Sciences and Microbiology, National Cheng Kung University, No. 1, University Road, Tainan City 701, Taiwan; Institute of Clinical Medicine, National Yang Ming Chiao Tung University, No. 155, Sec. 2, Linong St. Beitou Dist., Taipei City 112304, Taiwan; Department of Oncology, Taipei Veterans General Hospital, No. 201, Sec. 2, Shipai Rd., Beitou Dist., Taipei City 112201, Taiwan; Cancer and Immunology Research Center, National Yang Ming Chiao Tung University, No. 155, Sec. 2, Linong St. Beitou Dist., Taipei City 112304, Taiwan; Institute of Bioinformatics and Systems Biology, College of Engineering Bioscience, National Yang Ming Chiao Tung University, No. 75, Boai Street, East Dist., Hsinchu City 300, Taiwan; Cancer and Immunology Research Center, National Yang Ming Chiao Tung University, No. 155, Sec. 2, Linong St. Beitou Dist., Taipei City 112304, Taiwan; Center for Intelligent Drug Systems and Smart Bio-devices (IDS2B), National Yang Ming Chiao Tung University, No. 75, Boai Street, East Dist., Hsinchu City 300, Taiwan

**Keywords:** chemotherapy response, miRNA, multi-omics integration, machine learning, drug resistance

## Abstract

Chemotherapy remains a cornerstone of cancer treatment, yet therapeutic resistance continues to limit durable clinical responses. MicroRNAs (miRNAs) are key post-transcriptional regulators of gene expression that orchestrate diverse cellular processes implicated in drug sensitivity and resistance. However, the contribution of miRNA regulatory networks to chemotherapy response has not been systematically characterized using clinical patient cohorts. Here we present DrugMiR, an integrative multi-omics modeling framework that integrates drug response-associated miRNAs with experimentally validated target genes to identify regulatory signatures predictive of chemotherapy response. Using 1015 patient samples from The Cancer Genome Atlas, we identified differentially expressed miRNAs (DEmiRs) associated with response to five widely used chemotherapeutic agents (cisplatin, carboplatin, gemcitabine, paclitaxel, and fluorouracil) and constructed predictive models using multiple machine learning algorithms. DrugMiR achieved robust predictive performance, particularly for platinum-based therapies, with area under the receiver operating characteristic curves of 0.81 for cisplatin and 0.79 for carboplatin. Integration of validated miRNA targets consistently improved predictive performance while substantially enhancing biological interpretability. Integrative network and pathway analyses identified recurrent miRNA–target regulatory programs shared across chemotherapy cohorts, particularly involving the miR-200 family and target genes such as BMP2, ITGA3, EGFR, FGFR1, MET, and IL6, implicating growth factor signaling, focal adhesion, extracellular matrix remodeling, and adaptive survival pathways in chemotherapy response. Together, these findings establish patient-derived miRNA–target regulatory signatures as robust predictors of chemotherapy response and provide a multi-omics framework for elucidating molecular mechanisms underlying therapeutic sensitivity and resistance across diverse cancer types.

## Introduction

Chemotherapy remains one of the most widely used systemic treatments for cancer and continues to serve as the backbone of therapy across diverse malignancies, either as first-line treatment or in combination with surgery, radiotherapy, targeted therapy, and immunotherapy [1, 2]. Platinum compounds, fluoropyrimidines, taxanes, and nucleoside analogs remain indispensable components of standard-of-care regimens for many solid tumors [1–3]. However, drug resistance remains the primary obstacle to achieving durable clinical responses, substantially limiting treatment efficacy and contributing to tumor recurrence, metastasis, and poor patient survival [4, 5]. Drug resistance is a critical problem because it affected 90% chemotherapy failures that influence patient survival rate and also waste of medical resources [6]. Drug resistance is caused by a multifactorial process driven by the survival of tumor subpopulations with distinct genetic profiles. Following treatment exposure, resistance emerges through multiple mechanisms, including secondary mutations, epigenetic reprogramming, alterations of drug targets, activation of compensatory signaling pathways, and remodeling of the tumor microenvironment [4, 5, 7, 8]. Consequently, overcoming chemotherapy resistance remains one of the most urgent challenges in cancer research and precision medicine.

To prevent and overcome drug resistance, traditional research has largely focused on identifying genetic and epigenetic drivers to guide therapeutic strategies [9]. Despite these advances, a focus on individual genetic or epigenetic factors cannot fully explain the complexity of treatment failure. This limitation has prompted increasing interest in other omics dimensions. Among these, microRNAs (miRNAs) have emerged as a critical regulatory layer in the development of resistant phenotypes [10]. miRNAs are small, non-coding RNAs, typically 19–25 nucleotides in length that regulate gene expression at post-transcriptional level. They play essential roles in key biological processes, including cell cycle progression, cell survival, and apoptosis [11, 12]. By simultaneously modulating multiple target messenger RNAs (mRNAs), miRNAs act as key regulators that can reshape cellular responses to chemotherapy. For example, miR-21 directly suppresses the tumor suppressors PTEN and PDCD4, thereby attenuating chemotherapy-induced apoptotic signaling and promoting survival pathway activation [13]. Similarly, miR-125b regulates members of the BCL-2 family and influences mitochondrial apoptotic priming; its upregulation reduces drug sensitivity by inhibiting mitochondrial outer membrane permeabilization and downstream caspase activation [14]. Collectively, systematic analysis of miRNA–target regulatory networks provides a functional framework for understanding tumor behavior. It helps uncover resistance mechanisms that may remain undetected by conventional genomic sequencing approaches alone.

Building upon the recognition of miRNAs as key functional regulators of drug resistance, there is still a lack of computational frameworks that systematically incorporate miRNA expression into clinical drug response prediction models, particularly in a pan-cancer context. Currently, A few pioneering papers have integrated miRNA data with other omics features to improve prediction accuracy: Ding *et al.* [15] and Bomane *et al.* [16] incorporated miRNA expression but omitted broader multi-omics integration, while more recent integrated efforts by Ogunleye *et al.* [17] were restricted to breast cancer. Overall, these existing clinical models are often restricted to a single cancer type or emphasize predictive accuracy without sufficient functional interpretability. It is necessary to transform computational biomarkers into actionable biological insights. Consequently, developing a pan-cancer framework that integrates miRNA profiles from patient samples and offers functional validation remains an unmet need.

To address this, we developed DrugMiR, an integrative multi-omics modeling framework to predict drug response and uncover the underlying regulatory mechanisms across five major chemotherapies. To our knowledge, DrugMiR is among the first pan-cancer frameworks to prioritize differentially expressed miRNAs (DEmiRs) as a central regulatory feature layer and explicitly connect them with experimentally validated, response-associated target genes. Rather than treating miRNA and mRNA expression as independent omics blocks, DrugMiR reconstructs their regulatory relationship through miRNA–target interactions and evaluates three complementary feature configurations: DEmiRs, intersected target genes, and their integration. These complementary outputs allow the framework to assess the predictive contribution of the upstream miRNA regulatory layer, its downstream gene component, and the combined regulatory signature while also providing flexibility across different biological contexts and data availability. Furthermore, DrugMiR framework utilizing multiple machine learning (ML) models and hyperparameter optimization to achieve competitive performance while maintaining model interpretability and reproducibility. Beyond statistical prediction, the framework incorporates a functional interpretation layer that connects computational outputs with patient-derived multi-omics evidence through survival analysis, SHapley Additive exPlanations (SHAP)–based feature attribution, functional enrichment, and miRNA–gene regulatory network analysis. Together, these components enable DrugMiR to move beyond isolated biomarker identification toward the characterization of coordinated miRNA–target regulatory signatures associated with drug sensitivity and resistance, providing candidate response-associated signatures for further clinical investigation.

## Materials and methods

The analytical workflow of this study consists of four interconnected components, progressing from pan-cancer omics data collection to biomarker interpretation (Fig. 1). First, The Cancer Genome Atlas (TCGA) clinical drug response labels were harmonized with matched miRNA and gene expression profiles, while Cancer Cell Line Encyclopedia (CCLE) and GSE25204 datasets were collected for external validation. Second, differential expression analysis (DEA) was performed to identify drug response-associated DEmiRs and differential expression genes (DEGs), followed by integration of DEmiRs with experimentally validated target genes to generate DEmiR-only, intersected target gene, and integrated feature sets. Third, an integrative ML framework was used to compare single- and multi-omics feature sets, with models trained on the TCGA training cohort, evaluated on independent test sets, and externally validated. Finally, SHAP analysis, survival stratification, functional enrichment, and regulatory network reconstruction were applied to assess the biological and clinical relevance of the identified signatures.

**Figure 1:**
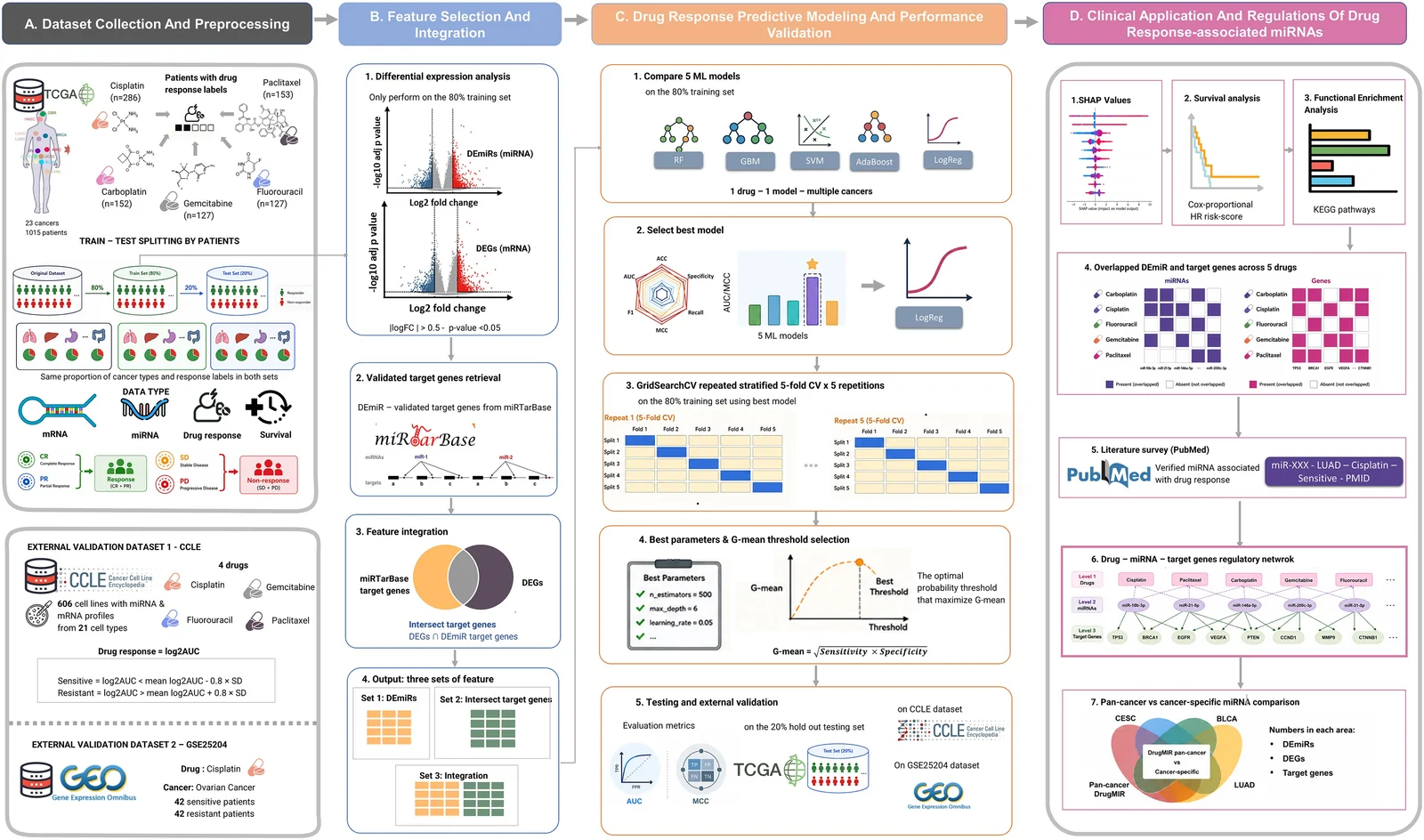
Overview of the DrugMiR analytical framework. (A) Integration of clinical drug response labels with TCGA multi-omics profiles; (B) identification of drug-associated gene and miRNA features using DEA followed by integration of experimentally validated miRNA target genes to generate single- and multi-omics feature sets; (C) evaluation of predictive performance through a integrative ML framework across single and multi-omics feature sets including training/testing dataset partitioning and independent validation; (D) downstream functional characterization, including SHAP interpretability, survival analysis, and miRNA–gene regulatory network reconstruction to assess biological relevance.

### Data source and data preprocessing

TCGA gene expression, miRNA expression, and clinical data were obtained from the University of California, Santa Cruz (UCSC) Xena platform (https://tcga.xenahubs.net). The initial pan-cancer cohort included 1197 patients across 28 cancer types and 152 drugs. Drug response annotations were obtained from Ding *et al.* [15]. Here, Complete Response (CR) and Partial Response (PR) were grouped as responders, while Stable Disease (SD) and Progressive Disease (PD) were grouped as non-responders. After retaining only patients with complete response labels, miRNA expression, and mRNA expression data, 1015 patients across 23 cancer types remained for analysis. The five drugs with the largest sample sizes—cisplatin, carboplatin, gemcitabine, paclitaxel, and fluorouracil—were selected for downstream analyses (Supplementary Fig. S1).

The preprocessing of omics data was conducted using R programming software, integrating TCGAbiolinks for data retrieval and miRBaseVersions.db [18] for miRNA annotation. The expression levels were quantified as Reads Per Million (RPM) for miRNA and Transcripts Per Million (TPM) for mRNA. Features and samples containing >20% zero or missing values were removed, resulting in 525–572 miRNAs and 24 235–24 733 genes per drug cohort prior to DEA. Expression matrices were subsequently transformed using ${\mathit{\log}}_2\left(x+1\right).$ The RNA-seq analysis pipeline utilized in this study was adopted from the methodology established by Lee *et al.* [19] and is consistent with established protocols for multi-omics patient stratification [20, 21].

### External validation datasets

External validation datasets were obtained from both the CCLE and Gene Expression Omnibus (GEO) databases. For CCLE, gene expression (TPM) and miRNA expression (RPM) profiles were downloaded from the DepMap portal (https://depmap.org/portal). Drug response data were obtained from the Genomics of Drug Sensitivity in Cancer (GDSC2) database [22]. Four drugs overlapping with TCGA (cisplatin, paclitaxel, gemcitabine, and fluorouracil) were included, while carboplatin was not presented in the CCLE dataset. Drug sensitivity labels were assigned using log₂AUC values, where samples with values below mean − 0.8 standard deviation (SD) were classified as sensitive and those above mean + 0.8 SD as resistant [23]. Intermediate samples were excluded. The final validation cohort included 190 cisplatin-, 211 fluorouracil-, 176 gemcitabine-, and 283 paclitaxel-treated cell lines from 21 cancer lineages (Supplementary Fig. S2A).

The GSE25204 dataset [24] consists of advanced-stage ovarian cancer patients treated with cisplatin, where patients were classified as cisplatin-sensitive or cisplatin-resistant based on surgical debulking status and time to relapse following first-line chemotherapy. Patients with intermediate clinical outcomes were excluded, resulting in 42 sensitive and 42 resistant samples (Supplementary Fig. S2B).

### Construction of training and testing datasets

For each drug-specific TCGA cohort, patients were partitioned into training and testing datasets using an 80:20 stratified split. To preserve both cancer-type composition and response distribution, stratification was performed on the combined variable *s* = (*c*,*r*) where (*c*) denotes the cancer type and (*r*) denotes the response class (responder or non-responder). For each stratum (s) containing (${n}_s$) samples, approximately ${n}_s^{train}=0.8{n}_s$ and ${n}_s^{test}=0.2{n}_s$ samples were assigned to the training and testing datasets, respectively. This strategy preserves the response proportion within each cancer type:


(1) \begin{equation*} {p}_k=\frac{R_k}{R_k+N{R}_k} \end{equation*}

where (${R}_k$) and ($N{R}_k$) denote the numbers of responders and non-responders in cancer type (*k*). Consequently, ${p}_k^{train}\approx{p}_k^{test}\approx{p}_k^{original}$ for all cancer types, ensuring that both datasets maintain the same cancer-type-specific response distribution as the original cohort. Strata containing only a single patient were retained in the training dataset to avoid instability during partitioning. The training set was subsequently used for DEA, feature selection, model development, and hyperparameter optimization, whereas the independent test set was reserved exclusively for final model evaluation (Supplementary Fig. S2C–G).

### Identification of drug response–associated gene and miRNA signatures as training features

DEA was performed exclusively on the training dataset using the limma package [25] to prevent information leakage. Responders and non-responders were compared separately for each of the five selected drugs, and the resulting transcriptomic changes were visualized using volcano plots. Because the primary objective of DEA was feature discovery for downstream ML rather than the identification of definitive biomarkers, significantly dysregulated candidates were selected using thresholds of *P*-value <.05 and a $\left| lo{g}_2\left( Fold\ Change\right)\right|>0.5$. This relatively permissive threshold was chosen to retain potentially informative molecular features for subsequent modeling. To evaluate selection stability, DEA was independently repeated within each of the 25 repeated cross-validation (CV) training partitions using the same criteria; these fold-specific feature sets were used only for stability assessment and did not replace the full-training feature set used for model development. To mitigate false positives, selected features were further evaluated through independent testing, external validation in CCLE and GEO datasets, experimentally validated miRNA–target interactions, literature-supported filtering, and downstream biological interpretation.

To incorporate biological regulatory information into the feature selection process, experimentally validated miRNA–target interactions were retrieved from miRTarBase [26] using the multiMiR R package [27]. DEGs were intersected with validated miRNA targets to generate a biologically relevant target gene set. Three feature configurations were subsequently constructed: (i) DEmiRs, (ii) intersected target genes, and (iii) integration. The intersected target-gene model was not intended as a miRNA-independent gene-only benchmark; rather, it represents the downstream component of the miRNA-centered regulatory signature, in which target-gene expression is used without the corresponding miRNA expression features.

### Drug response prediction framework

A pharmaco-omics dataset for a specific drug can be represented as:


(2) \begin{equation*} D={\left\{\left({x}_i,{y}_i\right)\right\}}_{i=1}^n\kern0.75em \end{equation*}

where ${x}_i\in{R}^p$ is a high-dimensional vector containing the molecular features (DEmiRs, intersected genes, or integrated profiles) for the *i*-th patient tumor and ${y}_i\in \left\{0,1\right\}$ represents the drug response status.

To identify the optimal predictive architecture for drug response, a comparative benchmarking framework was implemented using five ML algorithms representing both linear and non-linear classification approaches: Logistic Regression (LogReg), Support Vector Machine (SVM), Random Forest (RF), Gradient Boosting Machine (GBM), and Adaptive Boosting (AdaBoost). All models were implemented in Python using the scikit-learn library (v1.0.2) [28]. The five classifiers were first evaluated using fixed/default model configurations across the DEmiR, intersected target gene, and integrated feature settings to provide a consistent baseline comparison.

Following the comparative benchmarking, LogReg was further subjected to hyperparameter optimization on the training set using five repetitions of stratified five-fold CV and GridSearchCV [28]. The optimization evaluated the solver, regularization penalty, and inverse regularization strength [(C)] separately for each drug and feature setting. Standard scaling was incorporated within the CV pipeline so that preprocessing parameters were estimated exclusively from the corresponding training folds and subsequently applied to the held-out validation fold. For each candidate hyperparameter combination, the model was trained on four folds and evaluated on the remaining fold across the repeated CV procedure, and the configuration achieving the best CV performance was retained.

For the optimized model, predicted probabilities generated within each CV fold were used to construct ROC curves. The classification threshold was determined by maximizing the Geometric Mean (G-mean), defined as:


(3) \begin{equation*} G- mean=\sqrt{Sensitivity\times Specificity} \end{equation*}

The threshold corresponding to the maximum G-mean was selected as the optimal operating point because it balances sensitivity and specificity under class-imbalanced conditions [29]. The resulting threshold was subsequently applied to the independent test set and external validation datasets for final performance evaluation.

The primary evaluation metrics included the Area Under the ROC Curve (AUC) and the Matthews Correlation Coefficient (MCC), the latter providing a balanced evaluation in the presence of class imbalances:


(4) \begin{equation*} MCC=\frac{TP\times TN- FP\times FN}{\sqrt{\left( TP+ FP\right)\left( TP+ FN\right)\left( TN+ FP\right)\left( TN+ FN\right)}} \end{equation*}

To determine if improvements in AUC between feature configurations (e.g. DEmiR-only versus Integrated) were statistically significant, the non-parametric DeLong test was employed:


(5) \begin{equation*} Z=\frac{\hat{AU{C}_1}-\hat{AU{C}_2}}{\sqrt{\mathrm{Var}\left(\hat{AU{C}_1}\right)+\mathrm{Var}\left(\hat{AU{C}_2}\right)-2\mathrm{Cov}\left(\hat{AU{C}_1},\hat{AU{C}_2}\right)}} \end{equation*}

A *P*-value *<.05* was considered to indicate a significant improvement in predictive performance between feature sets or algorithms.

### Quantifying molecular feature contributions with SHapley additive exPlanations

To improve model interpretability, SHAP were applied to the optimized models using the independent test dataset. SHAP values quantify the contribution of individual features to model predictions and are defined as:


(6) \begin{equation*} {\mathrm{\phi}}_i\left(f,x\right)={\sum}_{S\subseteq N\setminus \left\{i\right\}}\frac{\left|S\right|!\left(M-\left|S\right|-1\right)!}{M!}\left[f\left(S\cup \left\{i\right\}\right)-f(S)\right] \end{equation*}

where *M* represents the total number of features, *S* is a subset of features excluding *i*, and *f(S)* denotes the model’s expected output for that subset. Within the DrugMiR framework, SHAP analysis was applied to the optimized models using held-out validation or independent test samples to quantify the contribution of individual features to drug-response prediction. Model-specific explainers were used according to classifier architecture, including LinearExplainer for logistic regression, TreeExplainer for tree-based models, and KernelExplainer for non-tree models. For each analysis, the corresponding preprocessed training data were used as the background/reference dataset. Features were ranked according to their mean absolute SHAP values, while the magnitude and direction of individual feature effects were visualized using SHAP beeswarm plots.

To further assess SHAP robustness, feature-ranking stability was evaluated using repeated CV, bootstrap resampling, and external-dataset concordance. Across five repetitions of five-fold CV (25 runs), SHAP values were calculated on held-out validation folds, with stability assessed by top-20 feature recurrence and Spearman rank correlation. Bootstrap analysis used 100 resampled TCGA training cohorts per drug and feature setting; models were refitted while retaining the selected features and optimized configuration, and SHAP rankings were compared with the final TCGA test-set ranking. External concordance was evaluated by applying TCGA-derived models to compatible CCLE features without additional feature selection and comparing SHAP rankings with the corresponding TCGA test-set rankings using Spearman correlation and top-20 overlap.

### Survival analysis

Overall survival (OS) data from TCGA were used to evaluate the prognostic relevance of DrugMiR-derived signatures. To provide a clinical baseline, Cox proportional hazards models were first constructed using age and sex only. Molecular risk models were then generated by incorporating DrugMiR features into the clinical baseline, resulting in three model configurations for each drug cohort: (i) clinical covariates only (age + sex), (ii) clinical covariates plus DEmiRs (age + sex + DEmiR), and (iii) clinical covariates plus integrated DrugMiR features (age + sex + DEmiR + intersected target genes). For models containing molecular features, Lasso-penalized Cox proportional hazards regression was used to reduce dimensionality and construct risk scores. The Lasso–Cox model was implemented using the glmnet R package [30] for feature selection and risk-score construction, with the optimal penalty parameter determined by 10-fold CV. Risk scores were calculated as:


(7) \begin{equation*} RiskScore={\sum}_{i=1}^k{\mathrm{\beta}}_i{X}_i \end{equation*}

where ${\mathrm{\beta}}_i$ represents the Lasso-derived coefficient for each prioritized feature and ${X}_i$ represents the corresponding relative expression value. Patients were stratified into high- and low-risk groups using the median risk score. Prognostic performance was assessed using Kaplan–Meier analysis, hazard ratios (HRs), concordance index (C-index), and log-rank tests.

### Functional enrichment analysis

To elucidate the biological mechanisms underlying drug responses, Kyoto Encyclopedia of Genes and Genomes (KEGG) pathway enrichment analysis was performed on the miRNAs identified as significant drivers of drug response. This analysis was conducted independently for each of the five selected drugs to capture drug response–associated regulatory mechanisms. Experimentally validated target genes for these miRNAs were retrieved from the miRTarBase database using the multiMiR R package [27]. The enrichment was executed using the clusterProfiler package [31] with an adjusted *P*-value cutoff of .05. By identifying the signaling pathways regulated by these key miRNAs across each drug, a functional link was established between miRNA-mediated gene regulation and the clinical transition between sensitive and resistant phenotypes.

### Construction of miRNA-target regulatory networks

To investigate the regulatory mechanisms underlying drug response, drug–miRNA–gene regulatory networks were constructed using the identified DEmiRs and their corresponding intersected target genes. The network construction incorporated two layers of biological validation. The first layer was implemented during feature selection, where target genes were restricted to experimentally validated miRNA targets obtained from miRTarBase that were also identified as DEGs in the training cohort. This filtering strategy ensured that only biologically relevant regulatory interactions active in the analyzed samples were retained. As a second layer of biological validation, a comprehensive literature survey was performed using the ncRNADrug database [23] to identify miRNAs with supported associations to drug response or resistance for the five drugs in specific cancer types. Only DEmiRs with published evidence linking them to drug response were included in the final network visualization. The resulting drug–miRNA–gene networks were constructed and visualized using the igraph package in R [32]. Network topology metrics, including node degree and connectivity patterns, were subsequently analyzed to identify key regulatory miRNAs and genes associated with drug response.

## Result

### Performance of miRNA and target gene–based drug response prediction

A comprehensive characterization of the pan-cancer cohort and the drug-specific feature-reduction process is provided in Supplementary Result 1.1, Supplementary Fig. S3, and Supplementary Tables S1–S11. Briefly, drug-specific cohorts with matched molecular profiles and clinical response information were partitioned into training and independent held-out test sets. DEA was performed exclusively on the training cohorts to identify drug response–associated DEmiRs and DEGs. The identified DEmiRs were mapped to experimentally validated target genes using miRTarBase, which were subsequently intersected with response-associated DEGs to derive the final intersected target-gene sets. Supplementary Figure S3A illustrates this stepwise sample partitioning and feature-reduction procedure using cisplatin as a representative example, while Table 1 summarizes the corresponding sample and feature counts across all five drug cohorts. The resulting DEmiR, intersected target gene, and integrated DEmiR–target gene feature sets were subsequently used for ML-based drug response prediction. 

**Table 1:** Summary of sample partitioning and stepwise molecular feature reduction across five drug cohorts in the DrugMiR framework.

	**Cisplatin**	**Carboplatin**	**Gemcitabine**	**Fluorouracil**	**Paclitaxel**
**A. Cohort composition**					
Total patients	285	151	126	126	153
Training set	237	132	105	107	130
^↳^ Responders	185	83	52	66	87
^↳^ Non-responders	52	49	53	41	43
Held-out test set	48	19	21	19	23
^↳^ Responders	40	14	11	13	18
^↳^ Non-responders	8	5	10	6	5
**B. Feature reduction**					
Initial miRNA features	572	570	548	525	563
Initial gene features	24 338	24 309	24 733	24 235	24 313
DEmiRs	55	24	20	17	20
DEGs	834	378	503	576	573
Validated targets of upregulated DEmiRs	7875	1633	2051	618	1593
Validated targets of downregulated DEmiRs	2181	1407	1152	1440	439
Intersected target genes	417	52	171	188	93
Integrated feature set	472	76	191	205	113

Predictive performance was first compared across five ML algorithms using three feature configurations: DEmiRs, intersected target genes, and integrated DEmiR–target gene features. Across 15 scenarios (5 drugs × 3 feature sets), LogReg showed the most stable performance, achieving the highest scores across all six evaluation metrics in 60% of settings (9/15; Supplementary Fig. S4 and Tables S12–S16). Although RF and SVM occasionally achieved the best performance for specific metrics, LogReg maintained balanced performance across accuracy, MCC, F1-score, sensitivity, and specificity. Based on this comparative benchmarking, LogReg was selected as the final classifier for subsequent drug- and feature-specific hyperparameter optimization, held-out test evaluation, and interpretability analyses.

The LogReg models were then optimized separately for each drug and feature configuration using repeated five-fold CV and GridSearchCV, with the selected hyperparameters reported in Supplementary Table S19. The optimized models were subsequently evaluated on the independent held-out test sets (Fig. 2; Supplementary Table S17). Intersected target gene-based models showed stable performance across all five drugs, with AUC values ranging from 0.66 to 0.78 (Fig. 2A–E). In contrast, the contribution of DEmiRs was drug-dependent. DEmiR-only models achieved the best performance for cisplatin (AUC = 0.81, MCC = 0.34) and carboplatin (AUC = 0.79, MCC = 0.53), outperforming the corresponding target gene models (DeLong test *P* < .05). However, DEmiR-only models were weaker for paclitaxel (AUC = 0.58) and fluorouracil (AUC = 0.60), suggesting that miRNA expression alone may be insufficient for these drugs.

**Figure 2:**
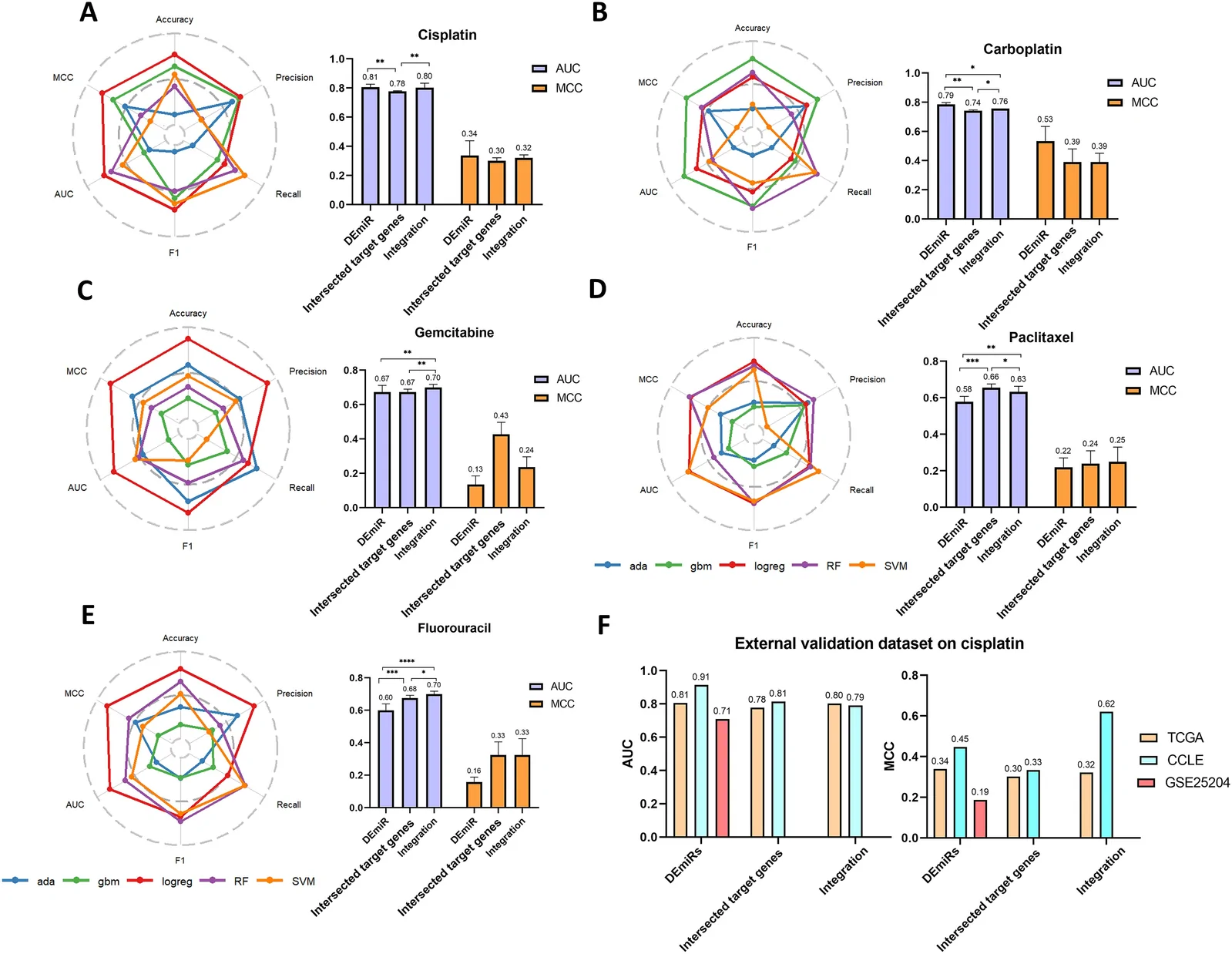
Comparative performance analysis of drug response prediction models (A–E) drug-specific performance evaluations for cisplatin (A), carboplatin (B), gemcitabine (C), paclitaxel (D), and fluorouracil (E). Each panel displays (left) radar plots comparing five ML algorithms (AdaBoost, GBM, LogReg, RF, and SVM) across multiple evaluation metrics and (right) bar charts showing the predictive performance of different feature sets (DEmiRs, intersected target genes, and integrated features); (F) external validation of cisplatin DrugMiR signatures across independent datasets. Prediction performance of DEmiR, intersected target gene, and integrated feature models was evaluated using the LogReg classifier in TCGA, CCLE, and GSE25204 cohorts, as measured by AUC and MCC. * *P* < .05; ** *P* < .01; *** *P* < .001, **** *P* < .0001.

Integration of DEmiRs and target genes provided a more balanced predictive framework. The integrated model achieved the highest AUC for gemcitabine and fluorouracil (both AUC = 0.70), while maintaining comparable performance for cisplatin (AUC = 0.80), carboplatin (AUC = 0.76), and paclitaxel (AUC = 0.63). DeLong analysis supported the benefit of integration for gemcitabine and fluorouracil, while target gene and integrated models significantly outperformed DEmiR-only models for paclitaxel (*P* < .05; Fig. 2C–E). These results suggest that combining miRNAs with their validated target genes improves or stabilizes prediction across drug cohorts.

To evaluate generalizability, optimized DrugMiR models were applied to independent CCLE and GEO datasets using the same TCGA-derived feature sets without additional feature selection. In CCLE, cisplatin showed the strongest external performance, with the DEmiR-only model achieving the highest AUC (0.914), the target gene model achieving the highest MCC (0.632), and the integrated model maintaining strong performance (AUC = 0.844, MCC = 0.563; Fig. 2F). For gemcitabine, integration achieved the highest AUC (0.805), whereas paclitaxel and fluorouracil were better supported by target gene-based models (Supplementary Table S17). In the independent GSE25204 ovarian cancer cohort, the cisplatin DEmiR model achieved an AUC of 0.71 and MCC of 0.19 (Fig. 2F). These findings indicate that DrugMiR-derived signatures retain predictive value in external datasets, although transferability varies by drug and feature configuration.

### Comparative evaluation of DrugMiR and existing platinum-drug response prediction methods

To contextualize DrugMiR performance, we compared our results with the TCGA-based platinum-drug response prediction study by Ding *et al.* [15], one of the few studies that systematically investigated cisplatin and carboplatin response using clinical patient cohorts across multiple cancer types. In the pan-cancer setting, Ding *et al.* reported that miRNA expression represented one of the most informative molecular layers for platinum-drug response prediction, achieving mean AUC values of ~0.69 for cisplatin and 0.60 for carboplatin across multiple cancer types. Using the same TCGA platinum-drug cohorts, DrugMiR achieved higher predictive performance, with DEmiR-only models reaching AUC values of 0.81 for cisplatin and 0.79 for carboplatin, and integrated DEmiR–target gene models achieving AUC values of 0.80 and 0.76, respectively (Table 2). 

**Table 2:** Performance comparison between DrugMiR and established models using original study patient populations. *Ding et al.* values were collected from the reported mean AUCs in Fig. S5. Comparisons should be interpreted qualitatively because the two studies employed different preprocessing, feature selection, model training, and validation procedures despite using the same TCGA clinical cohorts.

Drug	TCGA Cohort	Ding *et al.* (miRNA)	Ding *et al*. (mRNA)	DrugMiR (DEmiR)	DrugMiR (intersected target genes)	DrugMiR (integration)
Cisplatin	Pan-cancer	0.69	0.62	**0.81**	0.78	0.80
Carboplatin	Pan-cancer	0.60	0.60	**0.79**	0.74	0.76
Cisplatin	BLCA	0.60	0.58	0.53	0.66	**0.69**
Cisplatin	CESC	0.57	0.54	**0.73**	0.70	0.68
Cisplatin	LUAD	0.45	0.62	0.66	0.64	**0.68**
Carboplatin	UCEC	0.52	0.48	0.62	**0.67**	0.65

BLCA, bladder carcinoma; CESC, cervical squamous cell carcinoma; LUAD, lung adenocarcinoma; UCEC, uterine corpus endometrial carcinoma. Bold values indicate the highest AUC achieved among the compared models for each drug–cohort combination.

DrugMiR signatures were also evaluated in the cancer-specific platinum cohorts reported by Ding *et al.,* including cisplatin-treated bladder carcinoma (BLCA), cervical squamous cell carcinoma (CESC), lung adenocarcinoma (LUAD), and carboplatin-treated uterine corpus endometrial carcinoma (UCEC) cohorts. Despite differences in preprocessing, feature selection, and validation design, DrugMiR retained predictive capability in these individual cohorts (Table 2), supporting the potential transferability of pan-cancer drug response–associated signatures across tissue contexts.

### Survival stratification and SHAP-based interpretability of signature miRNAs

To further assess the clinical relevance and interpretability of DrugMiR signatures, survival stratification and SHAP analyses were performed using the selected feature sets (Supplementary Figs S5–S7, Supplementary Tables S20–S22, and Supplementary Results 1.2). Compared with the age- and sex-only clinical baseline, DEmiR-based models improved survival stratification across drug cohorts, and the addition of intersected target genes further strengthened the separation between high- and low-risk groups. SHAP analysis showed that miRNAs remained among the top predictive features despite representing a smaller fraction of the integrated feature space than target genes, with particularly strong contributions in cisplatin and carboplatin models. Repeated CV, bootstrap resampling, and external CCLE concordance further supported the reproducibility of major SHAP importance patterns, while indicating that the exact ordering of individual features may vary across resampling and datasets. These findings support the potential clinical relevance and biological interpretability of DrugMiR-derived chemotherapy response–associated signatures.

### Investigating drug resistance mechanism through miRNA-target regulatory networks and functional enrichment analysis

To investigate the biological relevance of DrugMiR signatures, overlap analysis, literature validation, KEGG enrichment, and regulatory network reconstruction were performed (Figs 3 and 4, Supplementary Table S23). Several DEmiRs were shared across multiple drug cohorts, particularly members of the miR-200 family, including miR-200a-3p, miR-200b-5p, miR-200c-3p, and miR-141-3p/5p, which were recurrently identified across platinum-based therapies, gemcitabine, and paclitaxel. Additional recurrent miRNA families included miR-10 and miR-181. Overlap was also observed among intersected target genes, including BMP2, ITGA3, TRPS1, EGFR, FGFR1, IL6, KLF6, LDLR, MET, and MYB, suggesting convergence of distinct chemotherapy agents on shared regulatory programs (Fig. 3A).

**Figure 3:**
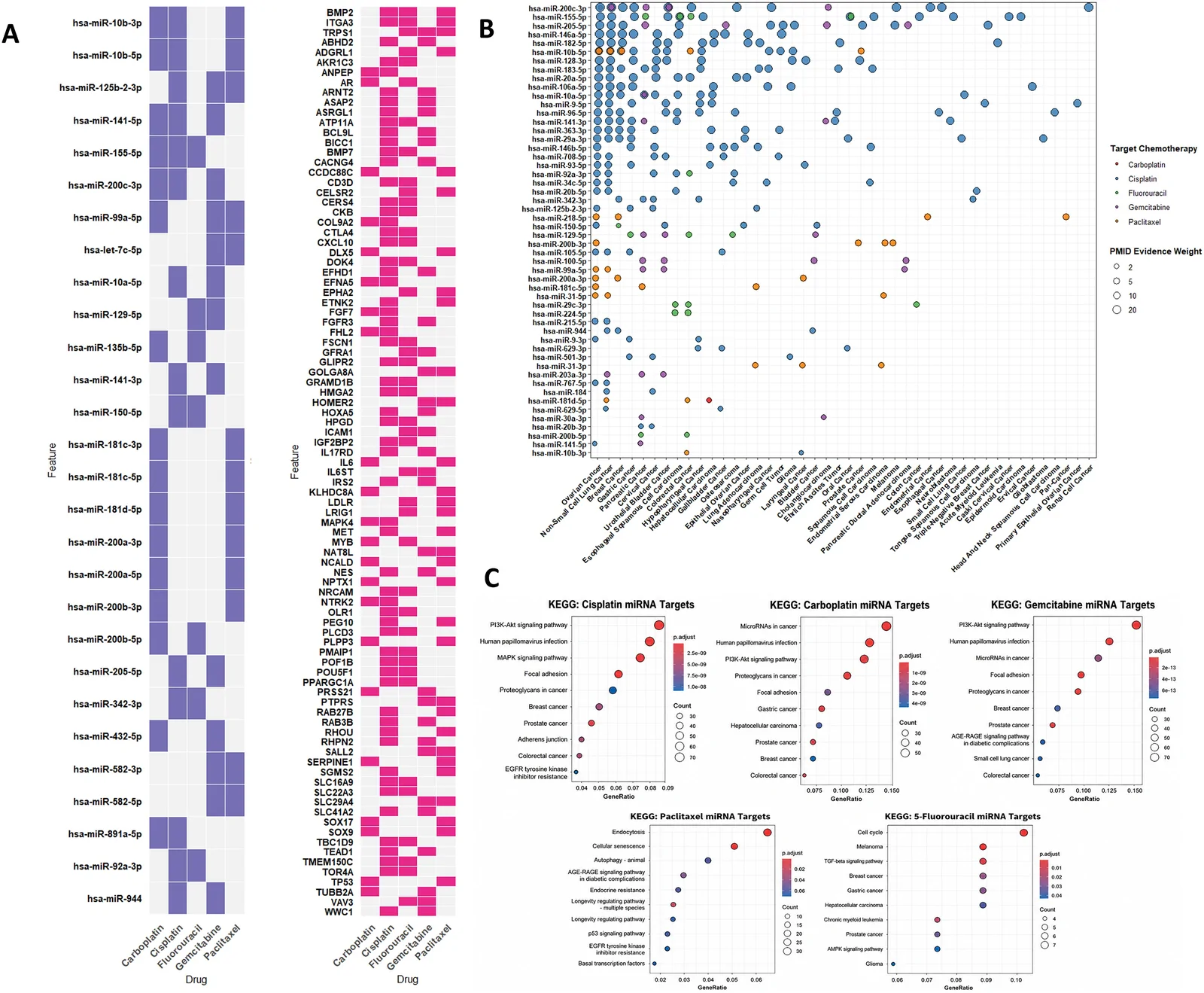
Multi-omics mapping and cross-cancer literature support for candidate drug response signatures. (A) Binary heatmap illustrating the overlapping landscape of statistically significant DEmiRs across five drugs. Filled cells indicate the presence of a specific DEmiR in the corresponding drug model or a validated downstream target-gene feature retained in the modeling workflow. (B) Bubble-dot distribution plot mapping the density of independent literature evidence supporting the identified candidate miRNAs in relation to drug response across various human malignancies. The *y*-axis indexes the candidate miRNAs, and the *x*-axis denotes specific cancer types. The bubble colors correspond to the targeted chemotherapy agents, while the bubble size represents the number of supporting publications (PMID), quantifying the total number of peer-reviewed studies validating each specific miRNA–drug–cancer axis. (C) KEGG pathway enrichment analysis of experimentally validated target genes associated with DEmiRs for each drug. Dot position indicates the GeneRatio shown on the *x*-axis, dot size represents gene count, and the marker scale represents adjusted *P*-value. Only pathways meeting an adjusted *P*-value threshold of <.05 are shown.

**Figure 4:**
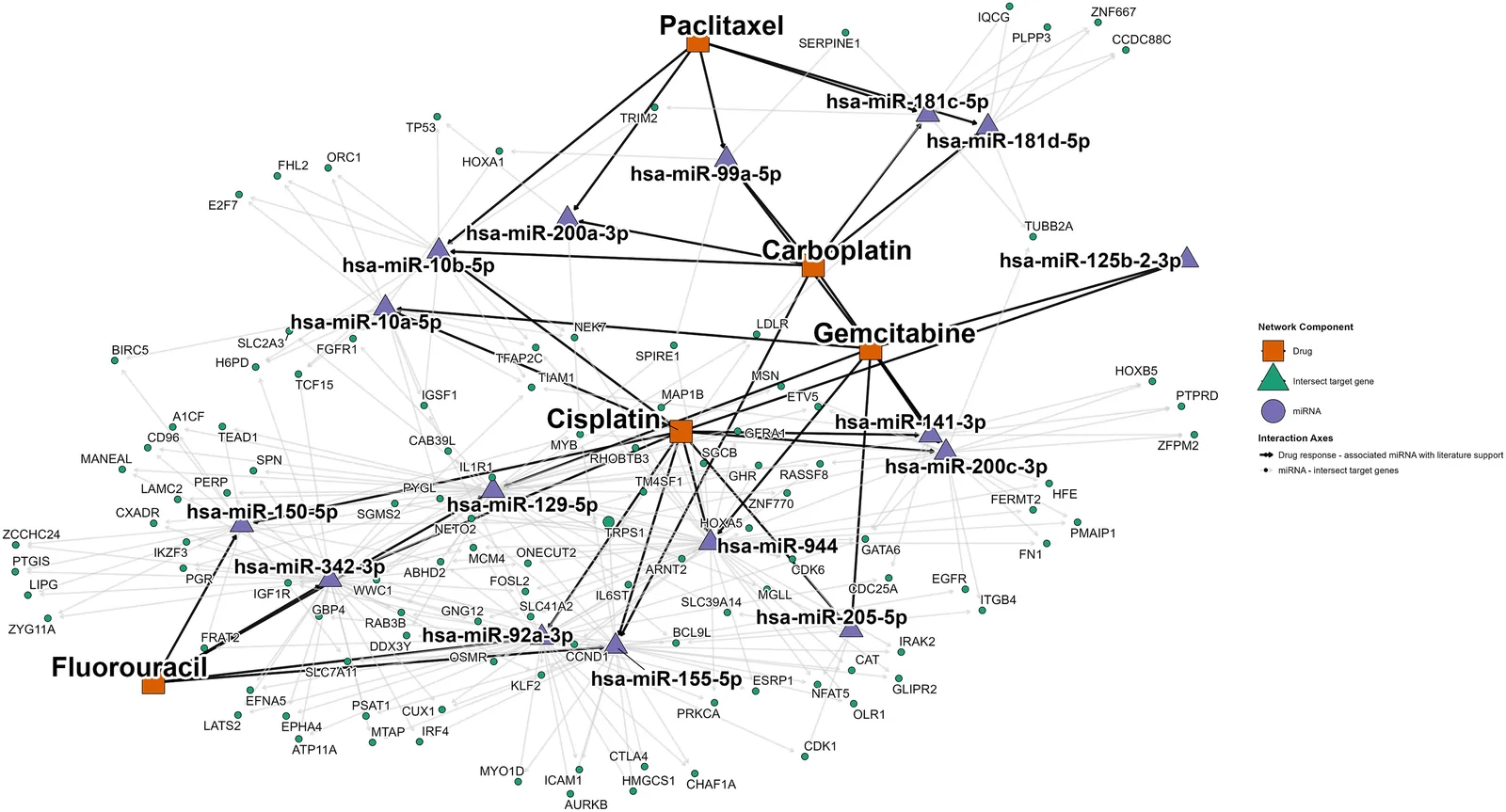
Integrative drug–miRNA–gene network analysis of drug response–associated signatures across five drugs. The interactome illustrates a unified three-layer biological regulatory network mapping the architectural cross-talk between chemotherapeutic agents, candidate miRNAs, and downstream functional intersected target genes. Rectangular shapes delineate specific drug response–associated landscapes, while triangles and circles represent DEmiRs and intersected target genes, respectively.

Literature validation using ncRNADrug and manually curated publications further supported the relevance of these signatures (Fig. 3B, Supplementary Table S23). MiRNAs supported by at least two independent studies were retained for downstream analysis, and the resulting evidence map showed that multiple prioritized miRNAs, particularly miR-200 family members, have been repeatedly associated with chemotherapy response across cancer types. KEGG enrichment analysis of experimentally supported DEmiR target genes intersected with DEGs revealed both shared and drug-specific pathways (Fig. 3C). Cisplatin, carboplatin, and gemcitabine were commonly enriched in PI3K-Akt signaling, focal adhesion, proteoglycans in cancer, and miRNAs in cancer, involving recurrent genes such as BMP2, ITGA3, EGFR, FGFR1, MET, and IL6. In contrast, paclitaxel-associated targets were enriched in endocytosis, cellular senescence, autophagy, and longevity-regulating pathways, whereas fluorouracil-associated targets were linked to cell cycle regulation, TGF-beta signaling, AMPK signaling, and metabolic pathways.

Finally, an integrative drug–miRNA–gene network was reconstructed using literature-supported miRNAs and their shared target genes (Fig. 4). The miR-200 family formed the dominant module, with miR-200a-3p, miR-200c-3p, and miR-141-3p linking cisplatin, carboplatin, gemcitabine, and paclitaxel through shared targets including EGFR, FERMT2, FN1, and ITGB4. Additional modules included the miR-10 family, which connected multiple drugs through genes related to transcriptional regulation and cellular adaptation, and miR-181c-5p/miR-181d-5p, which linked gemcitabine and paclitaxel. Together, these findings suggest that chemotherapy response is shaped by recurrent miRNA hubs regulating partially overlapping target gene networks.

### Comparative validation of DrugMiR biomarkers across independent TCGA cohorts

To evaluate the transferability of pan-cancer biomarkers to specific clinical contexts, DEA was performed on three independent cisplatin-treated TCGA cohorts: CESC (*n* = 62), BLCA (*n* = 41), and LUAD (*n* = 42) (Fig. 5A and B). Among these cohorts, CESC exhibited the greatest similarity to the pan-cancer cisplatin signature, sharing three of the nine pan-cancer DEmiRs, including miR-200c-3p, miR-141-3p, and miR-215-5p (Fig. 5C). Notably, miR-200c-3p and miR-141-3p were recurrently identified across multiple DrugMiR models and have been implicated as central regulators of platinum-drug response. Furthermore, GATA6 emerged as a shared target gene between the CESC-specific and pan-cancer analyses, linking cisplatin, carboplatin, and gemcitabine regulatory networks through interactions with miR-200c-3p and miR-363-3p (Fig. 4).

**Figure 5:**
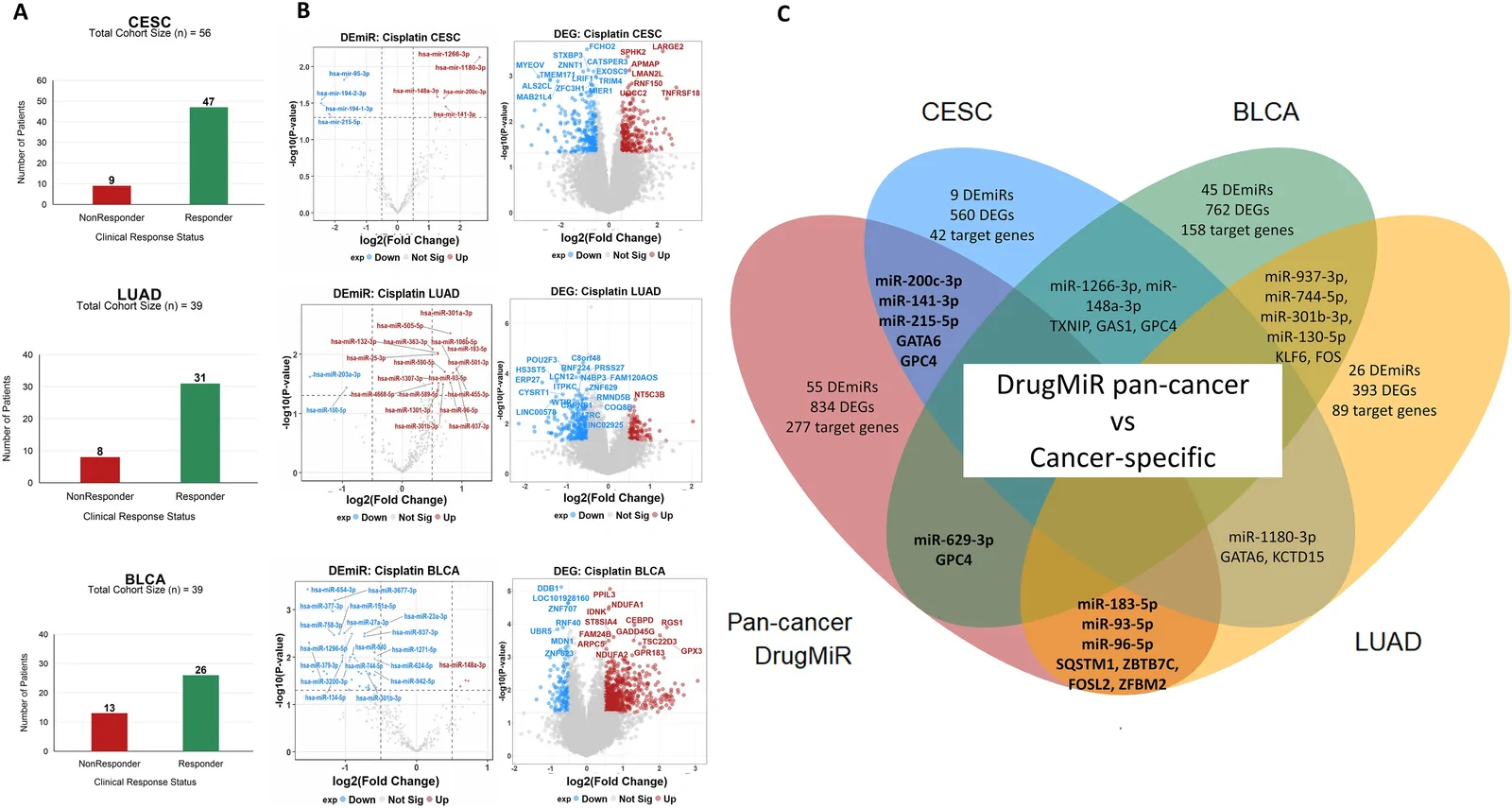
Validation of pan-cancer signatures on the cancer-specific cisplatin cohort. (A) Clinical response distribution for the CESC, LUAD, and BLCA validation cohort. (B) Volcano plots illustrating DEmiRs and DEGs in the cancers cohort. The significant DEGs were identified by using |log₂FC| > 0.5 and *P*-value <.05 (right: up-regulated, left: down-regulated). (C) Venn diagram demonstrating the overlap between cancer-specific results and the DrugMiR pan-cancer signatures. BLCA, bladder carcinoma; CESC, cervical squamous cell carcinoma; LUAD, lung adenocarcinoma; UCEC, uterine corpus endometrial carcinoma.

LUAD displayed overlap with the pan-cancer signature through miR-183-5p, miR-93-5p, and miR-96-5p, while target gene analysis identified ZFBM2 as a recurrent regulatory node previously linked to miR-200c-3p and miR-141-3p-mediated drug response mechanisms (Fig. 4). In contrast, BLCA shared fewer pan-cancer biomarkers and highlighted a potentially novel miR-629-3p–GPC4 regulatory axis associated with cisplatin response (Fig. 5C).

Importantly, several biomarkers prioritized by the pan-cancer framework, including miR-10a-5p, miR-34c-3p/5p, and miR-363-3p, were not consistently identified in the cancer-specific analyses. This finding highlights a key advantage of the DrugMiR approach: by integrating data across multiple cancer types, the framework increases statistical power and enables the identification of conserved drug-response regulators that may be overlooked in smaller individual cohorts. Collectively, these results demonstrate that DrugMiR captures both shared and tissue-specific mechanisms of chemotherapy response while preserving predictive relevance across diverse cancer contexts.

## Discussion and conclusions

The identification of robust molecular signatures for therapeutic response prediction remains a major challenge in precision oncology. Here, we developed DrugMiR, an integrative framework that integrates drug response–associated miRNAs and their experimentally validated target genes to predict chemotherapy response across five therapeutic agents. By combining DEA, ML, literature-supported target validation, and regulatory network interpretation, DrugMiR captures both upstream post-transcriptional regulation and downstream transcriptional programs associated with drug sensitivity and resistance.

A key finding is that the contribution of miRNAs to drug response prediction varies across therapeutic classes (Fig. 2). Platinum-based therapies, particularly cisplatin and carboplatin, demonstrated strong predictive performance using DEmiRs alone, suggesting that miRNA dysregulation captures a substantial proportion of the molecular determinants underlying platinum sensitivity and resistance. This is consistent with previous studies reporting miRNAs as highly informative predictors of platinum-drug response [15]. Nevertheless, integrating DEmiRs with target genes generally improved or stabilized performance across all drugs, highlighting the complementary value of regulatory and transcriptomic information.

Beyond prediction, DrugMiR provides a practical and interpretable strategy for chemotherapy response modeling by evaluating three complementary feature configurations—DEmiRs, intersected target genes, and their integration—together with repeated CV and model optimization. This design is particularly suitable for relatively limited drug-specific clinical cohorts while preserving biological interpretability through SHAP analysis, survival stratification, pathway enrichment, literature validation, and miRNA–gene network reconstruction. Recent pathway-informed Transformer and graph-based approaches have demonstrated the value of modeling complex multi-omics and regulatory relationships [33–38]. DrugMiR adopts a complementary strategy by explicitly constructing biologically traceable miRNA–target features from patient-derived expression data and experimentally validated interactions. This regulatory-centered design enables predictive signatures to be directly linked to chemotherapy-response mechanisms and recurrent miRNA regulators across drug cohorts.

The biological interpretation of DrugMiR signatures consistently highlighted members of the miR-200 family as central regulators of chemotherapy response. The recurrent identification of miR-200a, miR-200b, miR-200c, and miR-141 across multiple drug cohorts aligns with their established roles in suppressing EMT and maintaining drug sensitivity through regulation of ZEB1/2 signaling. Previous studies have demonstrated that miR-200c restores cisplatin sensitivity through inhibition of RRM2 and AKT2 [39, 40]. while miR-200b contributes to platinum sensitivity by suppressing stemness-associated pathways [41, 42]. Consistent with these observations, DrugMiR identified members of the miR-200 family as drug response association biomarkers across cisplatin, carboplatin, and gemcitabine cohorts (Figs 3 and 4), suggesting conserved roles in chemotherapy response across cancer types.

Cancer-specific validation further supported the partial transferability of pan-cancer DrugMiR signatures. Among the evaluated cisplatin-treated cohorts, CESC showed the strongest predictive performance and the highest overlap with pan-cancer features, whereas BLCA and LUAD showed lower overlap and weaker predictive performance These differences indicate that pan-cancer performance likely reflects a combination of shared chemotherapy-response mechanisms and cancer type–specific molecular characteristics. Accordingly, the applicability of a pan-cancer signature to an individual cancer type may depend on the extent to which the underlying regulatory programs are conserved within that tumor context. At the same time, the pan-cancer framework recovered candidate regulators that were not consistently detected in smaller cancer-specific cohorts, including miR-10a, miR-34c, and miR-363, which have been linked to platinum-drug response through PTEN, PIK3CA [43, 44], ATG4B, and HMGA2-related signaling [45–48]. These findings suggest that pan-cancer integration can improve statistical power for identifying shared response–associated mechanisms but should not be interpreted as implying that the same molecular determinants contribute equally across all cancer types.

Pathway and network analyses indicated that chemotherapy response is shaped by both shared and drug-specific regulatory programs. Cisplatin, carboplatin, and gemcitabine showed enrichment in PI3K-Akt signaling, focal adhesion, proteoglycans in cancer, and growth factor–related pathways, suggesting conserved survival and adhesion-associated mechanisms. In contrast, paclitaxel and fluorouracil were associated with more drug-specific processes, including autophagy, cellular senescence, cell cycle regulation, TGF-beta signaling, AMPK signaling, and metabolic pathways.

Several limitations should be acknowledged. Although external validation was performed using independent CCLE and GEO datasets, the primary discovery framework remains based on TCGA clinical cohorts. In addition, because the pan-cancer analysis combines biologically heterogeneous tumor types, part of the observed predictive signal may reflect cancer type–specific molecular differences rather than universally shared chemotherapy-response mechanisms. Although the cancer-specific analyses provide evidence that some pan-cancer signatures are transferable, their performance varied across tumor types, and larger cancer-specific cohorts will be needed to disentangle shared drug-response biology from tissue-specific effects. Additional validation in larger independent patient cohorts will be necessary to establish the generalizability of the identified biomarkers. Furthermore, the current study focuses on miRNA–mRNA regulatory interactions and does not incorporate additional molecular layers such as DNA methylation, copy number alterations, proteomics, or single-cell profiling. Finally, while the identified regulatory networks and pathways are supported by literature evidence and experimentally validated miRNA–target interactions, the inferred mechanisms remain computational and require experimental validation.

In conclusion, DrugMiR provides an interpretable multi-omics framework for chemotherapy response prediction using drug response-associated miRNAs and validated target genes. The framework captures both regulatory and transcriptional mechanisms associated with therapeutic response while maintaining biological interpretability. Its predictive value in cancer-specific cohorts suggests that pan-cancer DrugMiR signatures may reflect conserved chemotherapy response mechanisms, providing a foundation for future therapeutic stratification and biomarker discovery studies.

**Key Points**

- DrugMiR is an explainable multi-omics framework that integrates drug response–associated miRNAs with experimentally validated target genes to predict responses to five widely used chemotherapeutic agents across pan-cancer cohorts. The framework incorporates multiple machine learning (ML) algorithms with hyperparameter optimization, supports flexible predictive outputs, and provides integrative biological interpretation through miRNA–target regulatory networks.
- DrugMiR achieved robust predictive performance in TCGA pan-cancer cohorts, with DEmiR-based models showing the highest performance for platinum-based therapies, including cisplatin (AUC = 0.81, MCC = 0.34) and carboplatin (AUC = 0.79, MCC = 0.53).
- External validation demonstrated the transferability of DrugMiR signatures across various datasets, with cisplatin response prediction achieving AUC = 0.91 in Cancer Cell Line Encyclopedia (CCLE) using DEmiR features and AUC = 0.71 in the independent GSE25204 cohort, while the integrated DEmiR and intersected-target genes model achieved the highest CCLE MCC (0.62).
- Pan-cancer DrugMiR feature sets retained predictive value in TCGA cancer-specific platinum-drug cohorts, including BLCA, CESC, LUAD, and UCEC, suggesting that some drug response-associated regulatory signatures are transferable across cancer types.
- Network analysis identified the miR-200 family as a dominant regulatory module, with miR-200a-3p, miR-200c-3p, and miR-141-3p linking cisplatin, carboplatin, gemcitabine, and paclitaxel through shared target genes including EGFR, FERMT2, FN1, and ITGB4. These targets are associated with growth regulation, cell adhesion, and extracellular matrix–related signaling, suggesting that recurrent miRNA–gene regulatory hubs contribute to chemotherapy response across drug cohorts.

## Supplementary Material

Supplementary_Tables_S1-S23_revision_bbag560

## Data Availability

DrugMiR source code is available at https://github.com/tphuonglam/DrugMiR.
