# Supplementary material for: An integrative multi-omics modeling framework for chemotherapy response prediction through microRNA–target regulatory signatures
Source: Brief Bioinform. 2026 Oct 13;27(5):bbag560. doi: 10.1093/bib/bbag560 (PMC13660478; doi:10.1093/bib/bbag560)
Supplement: Supplementary file 1 [file supplementary_tables_s1-s23_revision_bbag560.zip › Supplementary_Tables_S1-S23_revision_bbag560/Supplementary_Material_DrugMir_Revision_bbag560.docx]

**An Integrative Multi-Omics Modeling Framework for Chemotherapy Response Prediction through microRNA–Target Regulatory Signatures**

1 **Supplementary Results**
1.1 **Transcriptomic feature selection for drug response prediction across five drugs**

After matching drug-response information with the corresponding miRNA and gene-expression profiles, the drug-specific paired cohorts comprised 285 patients for cisplatin, 151 for carboplatin, 153 for paclitaxel, and 126 each for gemcitabine and fluorouracil. These cohorts were further partitioned into stratified training and independent held-out test sets, with feature selection performed exclusively on the training data. For example, the cisplatin cohort included 237 training patients (185 responders and 52 non-responders) and 48 held-out test patients (40 responders and 8 non-responders), as illustrated in **Figure S3A**. Class distributions varied, with cisplatin showing the highest proportion of non-responders, whereas gemcitabine exhibited a nearly balanced ratio between responders and non-responders. To ensure that the input features for the ML framework possess robust biological relevance, a supervised feature selection process was conducted using DEA on miRNAs and genes with a threshold of $\left| log_{2\text{FC}} \right|>0.5$ and p-value < 0.05. The resulting DEmiRs and DEGs demonstrated substantial drug-specific variation, highlighting distinct regulatory programs underlying chemotherapy response **(Fig. S3B–F; Supplementary Tables S1–S10).** Repeated 5 × 5 cross-validation demonstrated moderate-to-high reproducibility of the selected feature sets, with mean overlap rates of 60.6–77.9% for DEmiRs and 66.7–77.4% for DEGs. Feature-level selection frequencies across the 25 resampling runs and BH-adjusted P-values are reported in **Supplementary Tables S1–S10**, while the overall concordance between nominal and adjusted P-value rankings and the cross-validation feature-set stability are summarized in **Supplementary Table S11**.

Cisplatin exhibited the most extensive transcriptomic alterations, with numerous significantly dysregulated miRNAs and genes, whereas fluorouracil displayed a more focused molecular signature characterized by a smaller number of highly dysregulated miRNAs. Across multiple drug cohorts, several members of the miR-200 family, including miR-141-3p, miR-200a-3p, miR-200b-5p, and miR-200c-3p, were consistently identified as upregulated in responders, particularly in cisplatin, carboplatin, and gemcitabine. Additional recurrent miRNAs included miR-148a-5p, miR-181c-5p, and miR-10b-3p/5p, suggesting the presence of conserved regulatory mechanisms shared among platinum-based therapies. In contrast, paclitaxel and fluorouracil displayed more distinct miRNA signatures. Paclitaxel was characterized by the enrichment of miR-181d-5p, miR-181c-5p, and miR-125b-2-3p, whereas fluorouracil showed a unique profile dominated by miR-129-5p, miR-142-3p, and miR-155-5p. At the gene level, most DEGs were drug-specific; however, several genes, including S100P, WFDC2, ST3GAL4, and FEZ1, were recurrently identified across multiple cohorts. Together, these findings indicate that chemotherapy response is governed by both shared regulatory programs, exemplified by the recurrent miR-200 family, and drug-specific transcriptomic signatures, providing biologically informed feature sets for subsequent machine learning analyses.

1.2 **Survival stratification and SHAP-based interpretability of significant miRNAs**

To evaluate the prognostic relevance of DrugMiR-derived drug response-associated signatures, survival stratification was performed using Cox-based risk score models with TCGA overall survival data (**Fig.S5**). For each of the five chemotherapy cohorts, three model configurations were compared: a clinical baseline model using age and sex only, a DEmiR-augmented model incorporating age, sex, and drug response-associated DEmiRs, and an integrated DrugMiR model combining age, sex, DEmiRs, and intersected target genes. This design enabled assessment of whether molecular features selected from responder versus non-responder comparisons provide additional prognostic information beyond basic clinical covariates.

Compared with the clinical baseline, the addition of DEmiRs improved survival stratification across multiple drug cohorts. For cisplatin (**Fig.S5A**), the age + sex model already separated patients into distinct survival groups (p = 1.15e-05, HR = 2.57), but adding DEmiRs further increased the significance of the separation (p = 1.04e-11, HR = 4.79), and the integrated DEmiR–target gene model produced the strongest risk-group separation (p =2.84e-16, HR = 7.58). A similar stepwise improvement was observed for carboplatin (**Fig.S5B**), where the age + sex model showed only modest prognostic separation (p = 0.00104, HR = 2.31), whereas the DEmiR-augmented and integrated models produced clearer separation between high-risk and low-risk groups (p = 9.04e-07, HR = 3.57). For gemcitabine (**Fig.S5C**), the clinical baseline showed limited separation (p = 0.37, HR = 1.24 (0.775-1.99)), but the addition of DEmiRs markedly improved the survival difference, with the integrated model further strengthening the prognostic signal (p = 1.92e-06, HR = 3.24). Paclitaxel and fluorouracil **(Fig. S5D-E)** also showed improved stratification after incorporating DrugMiR molecular features, with the integrated models producing the most distinct separation between high-risk and low-risk patients. Overall, these results show that DEmiRs selected from responder versus non-responder comparisons provide prognostic information beyond age and sex alone, while the addition of intersected target genes further enhances survival stratification. Because both DEmiRs and target genes were identified based on drug response status within each chemotherapy cohort, the improved survival separation suggests that DrugMiR-derived signatures are associated not only with drug response classification but also with clinically relevant outcome differences in drug-treated pan-cancer patients.

The molecular drivers underlying the predictive models were investigated using SHAP beeswarm analysis, which quantifies the contribution and directionality of individual features to model predictions (**Fig. S6**). Although the integrated feature sets contained substantially more intersected target genes than DEmiRs (**Table 1**), multiple miRNAs emerged among the highest-ranking predictive features across the five drug-specific models. In particular, the cisplatin and carboplatin models contained several highly ranked miRNA predictors, consistent with the strong predictive performance of the corresponding DEmiR-based models (Fig. 2A–B). For gemcitabine, paclitaxel, and fluorouracil, target genes constituted a larger proportion of the integrated feature space, but several miRNAs remained among the influential predictors, suggesting complementary contributions of miRNA and downstream target-gene expression to drug-response prediction.The persistence of these miRNA signals in the top-tier feature hierarchy indicates that they provide unique, non-redundant regulatory information essential for capturing therapeutic response complexity. This molecular evidence reinforces the predictive accuracy **(Fig. 2)** and distinct clinical outcomes observed in the integrated KM survival analyses **(Fig.S5).**

To assess whether SHAP-based interpretations were dependent on a particular model fit, sample partition, or dataset, feature-importance stability was evaluated using repeated cross-validation, bootstrap resampling, and external-dataset concordance (**Fig. S7; Tables S20–S22**). Across five repetitions of five-fold cross-validation, DEmiR-based models showed consistently strong ranking concordance, with mean Spearman correlations ranging from 0.70 to 0.84 across the five drugs, while intersected target-gene and integrated models showed more drug-dependent stability (Table S20). Feature-level heatmaps further demonstrated that several highly ranked predictors were repeatedly retained across cross-validation partitions (**Fig. S7**).

Bootstrap analysis using 100 resampled TCGA training cohorts provided an additional test of robustness to training-sample perturbation. DEmiR models showed moderate concordance, with mean Spearman correlations of 0.54–0.62, whereas intersected target-gene and integrated models showed correlations of 0.47–0.55 and 0.47–0.56, respectively (**Table S21**). The lower concordance in the larger feature spaces suggests greater sensitivity of correlated gene features to changes in training-sample composition.

External SHAP concordance was further evaluated in CCLE (**Table S22**). Strong cross-dataset agreement was observed for most evaluable settings, particularly for intersected target-gene models (ρ = 0.904–0.951) and integrated models (ρ = 0.909–0.946). Cisplatin DEmiRs also showed strong concordance (ρ = 0.802; top-20 overlap = 85%). DEmiR results for gemcitabine and fluorouracil were interpreted cautiously because only a small number of compatible features were available in CCLE.

Taken together, the repeated cross-validation, bootstrap, and external-dataset analyses indicate that DrugMiR identifies a reproducible core of influential predictors, while the exact ordering of SHAP-ranked features remains sensitive to training-sample composition and cohort context. Therefore, biological interpretation was focused on recurrent high-importance features rather than on the precise rank of individual predictors.

**2 Supplementary Figures**

**Figure S1.** Distribution of clinical response and non-response across cancer types in pan-cancer and five therapeutic cohorts.

**Figure S2**. Distribution of cancer types and drug response labels across training, testing, and external validation cohorts. (A) Drug response distributions in the CCLE validation cohorts. (B) Drug response distribution in the GSE25204 validation cohort. (C–G) Comparison of cancer-type composition and responder/non-responder proportions between the training and testing datasets for cisplatin (C), carboplatin (D), paclitaxel (E), gemcitabine (F), and fluorouracil (G). The stratified data partitioning strategy preserved both cancer-type representation and response-class distributions between training and testing cohorts, minimizing sampling bias and ensuring representative model evaluation.

**Figure S3.** Drug-specific cohort partitioning, feature reduction, and differential-expression profiles across five drugs. (A) Representative DrugMiR workflow for the cisplatin cohort, showing the total paired cohort, stratified training/held-out test split, initial miRNA and gene feature dimensions, training-set differential-expression analysis, experimentally validated miRNA target retrieval, intersection with DEGs, and construction of the final DEmiR, intersected target-gene, and integrated feature sets. (B–F) Volcano plots for cisplatin (B), carboplatin (C), gemcitabine (D), paclitaxel (E), and fluorouracil (F), showing significant DEmiRs (red), significant DEGs (blue), and non-significant features (gray) using |log₂FC| > 0.5 and p-value < 0.05

**Figure S4***.* Radar plots illustrating the performance of five machine learning algorithms—Ada (blue), GBM (green), LogReg (red), RF (purple), and SVM (orange)—across six key metrics of drug response prediction: Accuracy, Precision, Recall, F1-score, AUC, and MCC. The performance is stratified by input omic resource: (A) DEmiRs, (B) Intersected target genes, and (C) Integration. Within each row, metrics are presented independently for the Cisplatin, Carboplatin, Gemcitabine, Paclitaxel, and Fluorouracil cohorts.

**Figure S5**. Clinical covariate-adjusted prognostic validation of DrugMiR risk scores across pan-cancer drug cohorts. Kaplan–Meier curves illustrate overall survival stratification for five chemotherapy cohorts: (A) cisplatin, (B) carboplatin, (C) gemcitabine, (D) paclitaxel, and (E) fluorouracil. For each drug cohort, three risk-score configurations were evaluated across rows: clinical covariates only (age + sex), clinical covariates with DEmiRs (age + sex + DEmiR), and clinical covariates with integrated DrugMiR features (age + sex + DEmiR + target genes). Patients were stratified into low-risk and high-risk groups based on the median model-derived risk score. Log-rank p-values and hazard ratios are shown within each panel.

**Figure S6.** SHAP interpretability profiles illustrating the contribution of individual miRNAs and target genes to model predictions of drug response across (A) cisplatin, (B) carboplatin, (C) gemcitabine, (D) fluorouracil, and (E) paclitaxel in TCGA 20% hold out testing sets. Each point represents a patient sample. Color indicates the feature expression level (red: high, blue: low) and the x-axis represents the SHAP value (impact on model output)

**Figure S7.** Stability of SHAP feature-importance rankings across five repeats of five-fold cross-validation. (A) DEmiR-based models, (B) intersected target gene-based models, and (C) integrated DEmiR–target gene models across cisplatin, carboplatin, gemcitabine, paclitaxel, and fluorouracil. For each drug and feature setting, SHAP values were calculated on the held-out validation fold for each of the 25 cross-validation models, and features were ranked according to their mean absolute SHAP values. Heatmap colors indicate SHAP rank across the 25 runs, with darker colors representing higher feature importance and light gray indicating that a feature was not ranked among the top 20 in that run.

**3 Supplementary Tables**

**Table S1-S10. Lists of DEGs and DEmiRs of the five drugs cisplatin, carboplatin, gemcitabine, paclitaxel, fluorouracil**

Please find the excel attachments for the detailed lists.

**Table S11. Cross-validation stability and P-value ranking concordance of DEmiR and DEG feature selection across five drugs.**

| **Drug** | | **Feature type** | **Full-training features, n** | **Top-N overlap: P vs adj.P, n/N (%)** | **Mean features per run** | **Mean overlap with full-training set, n (%)** |
| --- | --- | --- | --- | --- | --- | --- |
| Cisplatin | DEmiRs | | 55 | 53/55 (96.4%) | 54.64 | 42.84 (77.9%) |
|  | DEGs | | 983 | 983/983 (100.0%) | 1004.64 | 761.12 (77.4%) |
| Carboplatin | DEmiRs | | 24 | 23/24 (95.8%) | 26.04 | 15.44 (64.3%) |
|  | DEGs | | 438 | 334/438 (76.3%) | 455.16 | 292.12 (66.7%) |
| Gemcitabine | DEmiRs | | 20 | 8/20 (40.0%) | 20.56 | 12.12 (60.6%) |
|  | DEGs | | 568 | 566/568 (99.6%) | 622.68 | 384.00 (67.6%) |
| Fluorouracil | DEmiRs | | 17 | 17/17 (100.0%) | 18.76 | 11.12 (65.4%) |
|  | DEGs | | 729 | 729/729 (100.0%) | 687.16 | 489.88 (67.2%) |
| Paclitaxel | DEmiRs | | 20 | 20/20 (100.0%) | 21.04 | 15.56 (77.8%) |
|  | DEGs | | 689 | 681/689 (98.8%) | 730.4 | 482.40 (70.0%) |

**Table S12.** Performance of cisplatin drug response prediction across five different ML models

| **Model** | **Feature Set** | **Accuracy** | **Precision** | **Recall** | **F1-Score** | **AUC** | **MCC** |
| --- | --- | --- | --- | --- | --- | --- | --- |
| **LogReg** | DEmiRs | 0.793 | 0.834 | 0.923 | 0.875 | 0.763 | 0.31 |
|  | Intersected target genes | 0.807 | 0.862 | 0.901 | 0.879 | 0.765 | 0.401 |
|  | Integration | 0.803 | 0.868 | 0.887 | 0.876 | 0.765 | 0.406 |
| **Ada** | DEmiRs | 0.743 | 0.827 | 0.855 | 0.839 | 0.705 | 0.198 |
|  | Intersected target genes | 0.76 | 0.824 | 0.892 | 0.853 | 0.737 | 0.222 |
|  | Integration | 0.767 | 0.817 | 0.906 | 0.858 | 0.676 | 0.207 |
| **GBM** | DEmiRs | 0.783 | 0.833 | 0.907 | 0.868 | 0.712 | 0.256 |
|  | Intersected target genes | 0.777 | 0.805 | 0.946 | 0.869 | 0.697 | 0.159 |
|  | Integration | 0.783 | 0.806 | 0.954 | 0.873 | 0.695 | 0.151 |
| **RF** | DEmiRs | 0.767 | 0.796 | 0.945 | 0.863 | 0.754 | 0.119 |
|  | Intersected target genes | 0.81 | 0.824 | 0.966 | 0.888 | 0.766 | 0.284 |
|  | Integration | 0.81 | 0.823 | 0.967 | 0.888 | 0.762 | 0.295 |
| **SVM** | DEmiRs | 0.777 | 0.795 | 0.966 | 0.871 | 0.739 | 0.073 |
|  | Intersected target genes | 0.803 | 0.813 | 0.974 | 0.885 | 0.775 | 0.288 |
|  | Integration | 0.803 | 0.816 | 0.97 | 0.885 | 0.778 | 0.301 |

**Table S13.** Performance of carboplatin drug response prediction across five different ML models

| **Model** | **Feature Set** | **Accuracy** | **Precision** | **Recall** | **F1-Score** | **AUC** | **MCC** |
| --- | --- | --- | --- | --- | --- | --- | --- |
| **LogReg** | DEmiRs | 0.686 | 0.745 | 0.815 | 0.772 | 0.745 | 0.251 |
|  | Intersected target genes | 0.717 | 0.807 | 0.780 | 0.790 | 0.808 | 0.356 |
|  | Integration | 0.741 | 0.829 | 0.787 | 0.804 | 0.811 | 0.429 |
| **Ada** | DEmiRs | 0.644 | 0.743 | 0.762 | 0.737 | 0.684 | 0.220 |
|  | Intersected target genes | 0.717 | 0.761 | 0.847 | 0.800 | 0.715 | 0.305 |
|  | Integration | 0.675 | 0.750 | 0.795 | 0.767 | 0.760 | 0.204 |
| **GBM** | DEmiRs | 0.711 | 0.762 | 0.827 | 0.785 | 0.767 | 0.338 |
|  | Intersected target genes | 0.730 | 0.757 | 0.890 | 0.815 | 0.754 | 0.323 |
|  | Integration | 0.711 | 0.743 | 0.881 | 0.802 | 0.753 | 0.279 |
| **RF** | DEmiRs | 0.692 | 0.722 | 0.888 | 0.787 | 0.719 | 0.256 |
|  | Intersected target genes | 0.754 | 0.767 | 0.919 | 0.834 | 0.795 | 0.386 |
|  | Integration | 0.730 | 0.753 | 0.901 | 0.818 | 0.779 | 0.314 |
| **SVM** | DEmiRs | 0.650 | 0.687 | 0.881 | 0.763 | 0.724 | 0.069 |
|  | Intersected target genes | 0.771 | 0.782 | 0.919 | 0.843 | 0.776 | 0.443 |
|  | Integration | 0.753 | 0.778 | 0.890 | 0.827 | 0.745 | 0.405 |

**Table S14.** Performance of gemcitabine drug response prediction across five different ML models

| **Model** | **Feature Set** | **Accuracy** | **Precision** | **Recall** | **F1-Score** | **AUC** | **MCC** |
| --- | --- | --- | --- | --- | --- | --- | --- |
| **LogReg** | DEmiRs | 0.626 | 0.630 | 0.547 | 0.585 | 0.654 | 0.250 |
|  | Intersected target genes | 0.684 | 0.682 | 0.667 | 0.659 | 0.759 | 0.381 |
|  | Integration | 0.690 | 0.694 | 0.695 | 0.681 | 0.765 | 0.397 |
| **Ada** | DEmiRs | 0.581 | 0.568 | 0.569 | 0.564 | 0.597 | 0.161 |
|  | Intersected target genes | 0.652 | 0.679 | 0.600 | 0.629 | 0.732 | 0.311 |
|  | Integration | 0.652 | 0.662 | 0.638 | 0.639 | 0.695 | 0.313 |
| **GBM** | DEmiRs | 0.523 | 0.514 | 0.492 | 0.499 | 0.546 | 0.044 |
|  | Intersected target genes | 0.645 | 0.652 | 0.612 | 0.628 | 0.727 | 0.291 |
|  | Integration | 0.632 | 0.659 | 0.546 | 0.593 | 0.720 | 0.268 |
| **RF** | DEmiRs | 0.542 | 0.531 | 0.534 | 0.531 | 0.605 | 0.084 |
|  | Intersected target genes | 0.677 | 0.704 | 0.599 | 0.644 | 0.736 | 0.359 |
|  | Integration | 0.665 | 0.681 | 0.585 | 0.628 | 0.741 | 0.329 |
| **SVM** | DEmiRs | 0.561 | 0.559 | 0.441 | 0.491 | 0.612 | 0.117 |
|  | Intersected target genes | 0.703 | 0.748 | 0.615 | 0.666 | 0.778 | 0.420 |
|  | Integration | 0.710 | 0.758 | 0.615 | 0.671 | 0.773 | 0.432 |

**Table S15**. Performance of paclitaxel drug response prediction across five ML models

| **Model** | **Feature Set** | **Accuracy** | **Precision** | **Recall** | **F1-Score** | **AUC** | **MCC** |
| --- | --- | --- | --- | --- | --- | --- | --- |
| **LogReg** | DEmiRs | 0.746 | 0.764 | 0.917 | 0.829 | 0.741 | 0.347 |
|  | Intersected target genes | 0.809 | 0.851 | 0.851 | 0.851 | 0.867 | 0.537 |
|  | Integration | 0.803 | 0.843 | 0.851 | 0.847 | 0.873 | 0.519 |
| **Ada** | DEmiRs | 0.683 | 0.766 | 0.792 | 0.771 | 0.668 | 0.252 |
|  | Intersected target genes | 0.746 | 0.778 | 0.880 | 0.824 | 0.789 | 0.322 |
|  | Integration | 0.765 | 0.797 | 0.878 | 0.833 | 0.789 | 0.398 |
| **GBM** | DEmiRs | 0.676 | 0.763 | 0.837 | 0.780 | 0.652 | 0.214 |
|  | Intersected target genes | 0.790 | 0.799 | 0.936 | 0.858 | 0.823 | 0.451 |
|  | Integration | 0.765 | 0.784 | 0.902 | 0.836 | 0.818 | 0.386 |
| **RF** | DEmiRs | 0.739 | 0.770 | 0.921 | 0.828 | 0.686 | 0.351 |
|  | Intersected target genes | 0.796 | 0.793 | 0.949 | 0.860 | 0.764 | 0.500 |
|  | Integration | 0.784 | 0.782 | 0.954 | 0.855 | 0.764 | 0.455 |
| **SVM** | DEmiRs | 0.733 | 0.737 | 0.949 | 0.826 | 0.743 | 0.291 |
|  | Intersected target genes | 0.802 | 0.803 | 0.941 | 0.863 | 0.785 | 0.522 |
|  | Integration | 0.802 | 0.803 | 0.941 | 0.863 | 0.789 | 0.522 |

**Table S16**. Performance of fluorouracil drug response prediction across five different ML models

| Model | **Feature Set** | **Accuracy** | **Precision** | **Recall** | **F1-Score** | **AUC** | **MCC** |
| --- | --- | --- | --- | --- | --- | --- | --- |
| **LogReg** | DEmiRs | 0.697 | 0.752 | 0.802 | 0.769 | 0.757 | 0.352 |
|  | Intersected target genes | 0.704 | 0.773 | 0.762 | 0.763 | 0.745 | 0.363 |
|  | Integration | 0.689 | 0.771 | 0.725 | 0.743 | 0.778 | 0.345 |
| **Ada** | DEmiRs | 0.651 | 0.732 | 0.718 | 0.722 | 0.639 | 0.260 |
|  | Intersected target genes | 0.628 | 0.699 | 0.728 | 0.712 | 0.662 | 0.187 |
|  | Integration | 0.652 | 0.715 | 0.752 | 0.730 | 0.696 | 0.249 |
| **GBM** | DEmiRs | 0.629 | 0.702 | 0.755 | 0.721 | 0.657 | 0.188 |
|  | Intersected target genes | 0.644 | 0.693 | 0.789 | 0.734 | 0.664 | 0.209 |
|  | Integration | 0.689 | 0.714 | 0.846 | 0.767 | 0.696 | 0.324 |
| **RF** | DEmiRs | 0.682 | 0.711 | 0.860 | 0.775 | 0.718 | 0.283 |
|  | Intersected target genes | 0.651 | 0.685 | 0.834 | 0.745 | 0.689 | 0.219 |
|  | Integration | 0.660 | 0.686 | 0.856 | 0.756 | 0.728 | 0.239 |
| **SVM** | DEmiRs | 0.667 | 0.699 | 0.860 | 0.766 | 0.703 | 0.237 |
|  | Intersected target genes | 0.674 | 0.709 | 0.835 | 0.760 | 0.745 | 0.290 |
|  | Integration | 0.681 | 0.723 | 0.812 | 0.759 | 0.757 | 0.309 |

**Table S17**. Performance of the LogReg drug response prediction model optimized with fine-tuned hyperparameters across five different drugs

| **Drug** | **Feature Set** | **Accuracy** | **Precision** | **Recall** | **F1-Score** | **AUC** | **MCC** |
| --- | --- | --- | --- | --- | --- | --- | --- |
| Cisplatin | DEmiRs | 0.833 | 0.881 | 0.925 | 0.902 | 0.806 | 0.338 |
|  | Intersected target genes | 0.729 | 0.909 | 0.750 | 0.822 | 0.778 | 0.306 |
|  | Integration | 0.792 | 0.895 | 0.850 | 0.872 | 0.803 | 0.321 |
| Carboplatin | DEmiRs | 0.789 | 0.916 | 0.786 | 0.846 | 0.786 | 0.535 |
|  | Intersected target genes | 0.684 | 0.900 | 0.642 | 0.75 | 0.743 | 0.390 |
|  | Integration | 0.619 | 0.875 | 0.650 | 0.761 | 0.757 | 0.390 |
| Gemcitabine | DEmiRs | 0.571 | 0.571 | 0.727 | 0.640 | 0.672 | 0.135 |
|  | Intersected target genes | 0.714 | 0.727 | 0.727 | 0.727 | 0.672 | 0.427 |
|  | Integration | 0.619 | 0.636 | 0.636 | 0.636 | 0.7 | 0.236 |
| Paclitaxel | DEmiRs | 0.601 | 0.800 | 0.667 | 0.727 | 0.578 | 0.224 |
|  | Intersected target genes | 0.752 | 0.813 | 0.722 | 0.765 | 0.656 | 0.238 |
|  | Integration | 0.696 | 0.824 | 0.778 | 0.800 | 0.633 | 0.247 |
| Fluorouracil | DEmiRs | 0.556 | 0.667 | 0.400 | 0.500 | 0.600 | 0.158 |
|  | Intersected target genes | 0.667 | 0.700 | 0.700 | 0.700 | 0.675 | 0.325 |
|  | Integration | 0.667 | 0.700 | 0.700 | 0.700 | 0.675 | 0.325 |

**Table S18**. Performance of the LogReg drug response prediction model on CCLE external validation dataset across four different drugs

| **Drug** | **Feature Set** | **Accuracy** | **Precision** | **Recall** | **F1-Score** | **AUC** | **MCC** |
| --- | --- | --- | --- | --- | --- | --- | --- |
| Cisplatin | DEmiRs | 0.722 | 0.728 | 0.733 | 0.721 | 0.914 | 0.465 |
|  | Intersected target genes | 0.800 | 0.933 | 0.667 | 0.773 | 0.867 | 0.632 |
|  | Integration | 0.767 | 0.886 | 0.644 | 0.739 | 0.844 | 0.563 |
| Gemcitabine | DEmiRs | 0.585 | 0.684 | 0.457 | 0.545 | 0.576 | 0.204 |
|  | Intersected target genes | 0.554 | 0.683 | 0.314 | 0.429 | 0.757 | 0.169 |
|  | Integration | 0.554 | 0.683 | 0.314 | 0.429 | 0.805 | 0.169 |
| Paclitaxel | DEmiRs | 0.400 | 0.255 | 0.656 | 0.367 | 0.460 | 0.030 |
|  | Intersected target genes | 0.608 | 0.371 | 0.688 | 0.481 | 0.669 | 0.237 |
|  | Integration | 0.567 | 0.344 | 0.688 | 0.456 | 0.639 | 0.188 |
| Fluorouracil | DEmiRs | 0.500 | 0.581 | 0.360 | 0.444 | 0.455 | 0.037 |
|  | Intersected target genes | 0.611 | 0.667 | 0.600 | 0.632 | 0.770 | 0.224 |
|  | Integration | 0.600 | 0.658 | 0.580 | 0.616 | 0.750 | 0.203 |

**Table S19**. Hyperparameter Grid for Logistic Regression Optimization

| **Parameter** | **Values** |
| --- | --- |
| Solver–penalty | liblinear–L1, liblinear–L2, lbfgs–L2 |
| C | 0.001, 0.01, 0.1, 1, 10, 100 |
| Feature scaling | StandardScaler |
| Decision threshold | Optimized using G-mean |

**Table S20. Cross-validation stability of SHAP feature rankings assessed by Spearman correlation across three feature settings.**

| **Setting** | **Drug** | **Fold 1** | **Fold 2** | **Fold 3** | **Fold 4** | **Fold 5** | **Mean ± SD** |
| --- | --- | --- | --- | --- | --- | --- | --- |
| **DEmiRs** | Cisplatin | 0.71 | 0.85 | 0.89 | 0.85 | 0.87 | 0.84 ± 0.07 |
|  | Carboplatin | 0.82 | 0.78 | 0.80 | 0.57 | 0.76 | 0.75 ± 0.10 |
|  | Gemcitabine | 0.76 | 0.76 | 0.71 | 0.82 | 0.74 | 0.76 ± 0.04 |
|  | Paclitaxel | 0.74 | 0.54 | 0.76 | 0.70 | 0.74 | 0.70 ± 0.09 |
|  | Fluorouracil | 0.75 | 0.51 | 0.75 | 0.75 | 0.82 | 0.72 ± 0.12 |
| **Intersected target genes** | Cisplatin | 0.79 | 0.77 | 0.79 | 0.64 | 0.75 | 0.75 ± 0.06 |
|  | Carboplatin | 0.60 | 0.58 | 0.25 | 0.55 | 0.59 | 0.51 ± 0.15 |
|  | Gemcitabine | 0.44 | 0.68 | 0.61 | 0.70 | 0.67 | 0.62 ± 0.11 |
|  | Paclitaxel | 0.71 | 0.74 | 0.77 | 0.77 | 0.62 | 0.72 ± 0.06 |
|  | Fluorouracil | 0.56 | 0.53 | 0.61 | 0.63 | 0.58 | 0.58 ± 0.04 |
| **Integration** | Cisplatin | 0.79 | 0.74 | 0.79 | 0.71 | 0.76 | 0.76 ± 0.04 |
|  | Carboplatin | 0.64 | 0.67 | 0.64 | 0.60 | 0.65 | 0.64 ± 0.02 |
|  | Gemcitabine | 0.34 | 0.64 | 0.58 | 0.59 | 0.64 | 0.56 ± 0.13 |
|  | Paclitaxel | 0.73 | 0.79 | 0.81 | 0.81 | 0.72 | 0.77 ± 0.04 |
|  | Fluorouracil | 0.55 | 0.51 | 0.54 | 0.51 | 0.53 | 0.53 ± 0.02 |

**Table S21. Bootstrap stability of SHAP feature rankings across drug-specific feature settings**

| **Setting** | **Drug** | **Bootstrap runs** | **Spearman, mean ± SD** | **Top-20 overlap with reference SHAP ranking, n** | **Top-20 overlap, %** |
| --- | --- | --- | --- | --- | --- |
| **DEmiRs** | Cisplatin | 100 | 0.62 ± 0.09 | 13.64 ± 1.28 | 68.2 ± 6.4 |
|  | Carboplatin | 100 | 0.60 ± 0.17 | 17.48 ± 0.80 | 87.4 ± 4.0 |
|  | Gemcitabine | 100 | 0.56 ± 0.18 | 20.00 ± 0.00* | 100.0 ± 0.0* |
|  | Fluorouracil | 100 | 0.54 ± 0.18 | 17.00 ± 0.00* | 85.0 ± 0.0* |
|  | Paclitaxel | 100 | 0.61 ± 0.15 | 20.00 ± 0.00* | 100.0 ± 0.0* |
| **Intersected target genes** | Cisplatin | 100 | 0.47 ± 0.06 | 8.74 ± 1.65 | 43.7 ± 8.2 |
|  | Carboplatin | 100 | 0.55 ± 0.10 | 13.26 ± 1.51 | 66.3 ± 7.5 |
|  | Gemcitabine | 100 | 0.49 ± 0.07 | 9.33 ± 2.04 | 46.7 ± 10.2 |
|  | Fluorouracil | 100 | 0.52 ± 0.07 | 10.81 ± 1.69 | 54.1 ± 8.4 |
|  | Paclitaxel | 100 | 0.54 ± 0.08 | 11.03 ± 1.87 | 55.2 ± 9.4 |
| **Integration** | Cisplatin | 100 | 0.47 ± 0.06 | 8.52 ± 1.77 | 42.6 ± 8.8 |
|  | Carboplatin | 100 | 0.56 ± 0.09 | 12.27 ± 1.85 | 61.4 ± 9.3 |
|  | Gemcitabine | 100 | 0.49 ± 0.07 | 9.16 ± 2.05 | 45.8 ± 10.2 |
|  | Fluorouracil | 100 | 0.54 ± 0.06 | 9.83 ± 1.83 | 49.2 ± 9.1 |
|  | Paclitaxel | 100 | 0.52 ± 0.08 | 10.32 ± 1.92 | 51.6 ± 9.6 |

*Top-20 overlap was not considered informative when the complete feature set contained ≤20 features.

**Table S22. External concordance of SHAP feature-importance rankings between TCGA and CCLE**

| **Feature setting** | **Drug** | **Spearman ρ** | **Top-20 overlap with TCGA reference, n (%)** |
| --- | --- | --- | --- |
| DEmiRs | Cisplatin | 0.802 | 17/20 (85%) |
|  | Gemcitabine | −0.100 | 5/5(100%)* |
|  | Fluorouracil | 0.600 | 6/6(100%)* |
| Intersected target genes | Cisplatin | 0.917 | 11/20 (55%) |
|  | Gemcitabine | 0.950 | 13/20 (65%) |
|  | Fluorouracil | 0.904 | 11/20 (55%) |
|  | Paclitaxel | 0.951 | 16/20 (80%) |
| Integration | Cisplatin | 0.914 | 7/20 (35%) |
|  | Gemcitabine | 0.946 | 13/20 (65%) |
|  | Fluorouracil | 0.909 | 11/20 (55%) |
|  | Paclitaxel | 0.941 | 16/20 (80%) |

*Top-20 overlap was not considered informative when the complete feature set contained ≤20 features.

**Table S23. Literature-supported cisplatin response-associated miRNAs and their reported cancer types.**

Please find the excel attachments for the detailed lists.
